# Silver(I) Ions Ultrasensitive Detection at Carbon Electrodes—Analysis of Waters, Tobacco Cells and Fish Tissues

**DOI:** 10.3390/s90906934

**Published:** 2009-09-01

**Authors:** Sona Krizkova, Olga Krystofova, Libuse Trnkova, Jaromir Hubalek, Vojtech Adam, Miroslava Beklova, Ales Horna, Ladislav Havel, Rene Kizek

**Affiliations:** 1 Department of Chemistry and Biochemistry, Mendel University of Agriculture and Forestry, Zemedelska 1, CZ-613 00 Brno, Czech Republic; 2 Department Chemistry, Faculty of Science, Masaryk University, Kotlarska 2, CZ-611 37 Brno, Czech Republic; 3 Research Centre for Environmental Chemistry and Ecotoxicology, Faculty of Science, Masaryk University, Kotlarska 2, CZ-611 37 Brno, Czech Republic; 4 Department of Microelectronics, Faculty of Electrical Engineering and Communication, Brno University of Technology, Udolni 53, CZ-602 00 Brno, Czech Republic; 5 Department of Animal Nutrition and Forage Production, Mendel University of Agriculture and Forestry, Zemedelska 1, CZ-613 00 Brno, Czech Republic; 6 Department of Veterinary Ecology and Environmental Protection, Faculty of Veterinary Hygiene and Ecology, University of Veterinary and Pharmaceutical Sciences, Palackeho 1-3, CZ-612 42 Brno, Czech Republic; 7 Tomas Bata University, T.G. Masaryka 275, CZ-762 72 Zlin, Czech Republic; 8 Department of Plant Biology Faculty of Agronomy, Mendel University of Agriculture and Forestry, Zemedelska 1, CZ-613 00 Brno, Czech Republic

**Keywords:** silver, guppy (*Poecilia reticulata*), tobacco cells, ecotoxicology, voltammetry, miniaturized carbon electrodes

## Abstract

We used carbon paste electrodes and a standard potentiostat to detect silver ions. The detection limit (3 Signal/Noise ratio) was estimated as 0.5 μM. A standard electrochemical instrument microanalysis of silver(I) ions was suggested. As a working electrode a carbon tip (1 mL) or carbon pencil was used. Limits of detection estimated by dilution of a standard were 1 (carbon tip) or 10 nM (carbon pencil). Further we employed flow injection analysis coupled with carbon tip to detect silver(I) ions released in various beverages and mineral waters. During first, second and third week the amount of silver(I) ions releasing into water samples was under the detection limit of the technique used for their quantification. At the end of a thirteen weeks long experiment the content of silver(I) ions was several times higher compared to the beginning of release detected in the third week and was on the order of tens of nanomoles. In subsequent experiments the influence of silver(I) ions (0, 5 and 10 μM) on a plant model system (tobacco BY-2 cells) during a four-day exposition was investigated. Silver(I) ions were highly toxic to the cells, which was revealed by a double staining viability assay. Moreover we investigated the effect of silver(I) ions (0, 0.3, 0.6, 1.2 and 2.5 μM) on guppies (*Poecilia reticulata*). Content of Ag(I) increased with increasing time of the treatment and applied concentrations in fish tissues. It can be concluded that a carbon tip or carbon pencil coupled with a miniaturized potentiostat can be used for detection of silver(I) ions in environmental samples and thus represents a small, portable, low cost and easy-to-use instrument for such purposes.

## 1. Introduction

**Figure 1.**
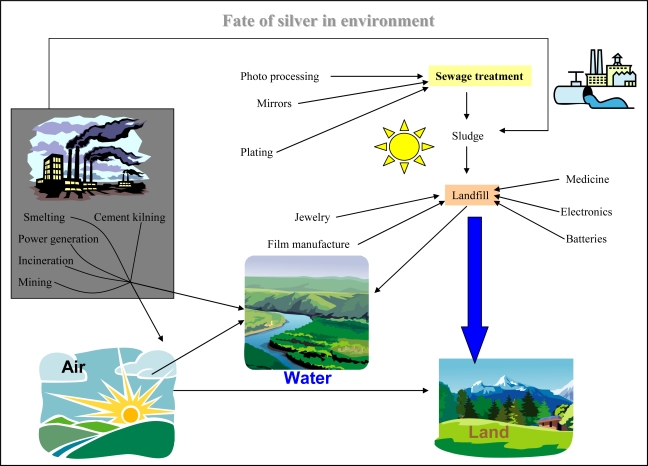
Scheme of silver(I) ion fate in the environment.

Due to many anthropogenic activities the environment has been polluted by a number of organic as well as inorganic compounds, and the concentrations of these undesirable and in most cases highly toxic substances have been steadily increasing. The mechanisms of their effects can be very heterogeneous. For these reasons the new procedures and technologies, not only to monitor the levels of contamination, but also to remediate the polluted environment are still developing. The heavy metal ions such as lead, cadmium, mercury, silver and their compounds are considered to be among the most toxic substances polluting all parts of environment [1–5]. The highest amounts of silver ions have been using in photographical industry (about 40% of the worldwide usage), followed by electrochemistry, medicine and others (Figure 1). They easily contaminate atmosphere as well as aquatic environment or soils [6]. Due to competing equilibria and kinetics of water hydrated silver ions, Ag^+^, this species may be also present in surface waters, which relates to the fact that Ag^+^ has been shown to be highly toxic to aquatic life [7]. Recently a number of papers reporting investigations on the influence of silver(I) ions on soil and water quality [8–11] and on the acute toxicity of these ions to aquatic organisms [12–20] have been published.

Determination of silver(I) ions in waters is difficult because the formation of a number of silver complexes with inorganic as well as organic compounds that depress the acute silver toxicity [15,18,19]. The determination of silver ions is usually carried out by atomic absorption spectrometry [21,22]. For enhancing the sensitivity of an analysis the pre-concentration of the silver ions in a sample is needed. These processes prolong the total time of the analysis as well as enhance the cost of such experiments [16,21,23]. Electrochemical methods are alternative analytic techniques that make the determination of silver ions possible in nM concentrations, mainly using carbon electrodes [24–30].

The aim of this work was to detect silver(I) ions by using various electrochemical instruments. Several carbon electrodes were tested for silver(I) determination. The optimized procedures were utilized for determination of silver(I) in various water samples, tobacco BY-2 cells and guppies treated with silver(I) ions.

## 2. Results and Discussion

### 2.1. Toxicity of Silver

**Figure 2.**
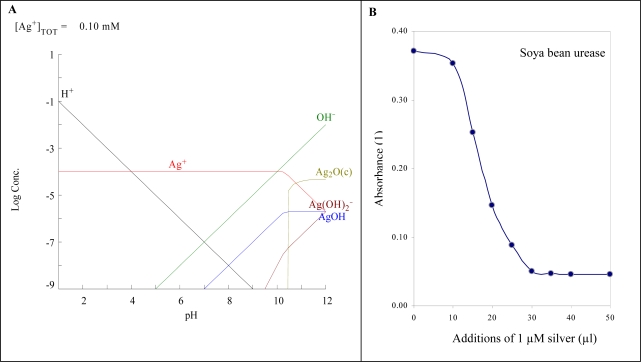
(A) Distribution diagram of silver(I) ions make by MEDUSA [37,38]. (B) Effect of silver(I) ions on enzyme urease isolated from soya beans.

Silver(I) ions in aquatic environment are stable over a wide range of pH values (Figure 2A). Only in alkaline solutions are hydroxides and hydroxide anions like AgOH and Ag(OH)_2_^−^ formed. There are many others compounds with which silver(I) ions can interact, forming many hardly soluble compounds, which do not pose a threat to organisms. The toxicity of silver ions is probably caused by their very good affinity for nucleic acids and proteins. The binding into an active site of the enzyme leads to the expressive inhibition of enzyme activity. This effect was observed by using of simple spectrometric test, in which we studied inhibition of urease activity by silver(I) ions (Figure 2B). Compared to cadmium(II) and lead(II) ions, silver(I) had the strongest inhibition effect [31]. Moreover an inhibition of enzyme activity was also shown in *in vivo* experiments [4,15,32–34].

### 2.2. Carbon Paste Electrode as a Tool for Determination of Silver(I) Ions

**Figure 3.**
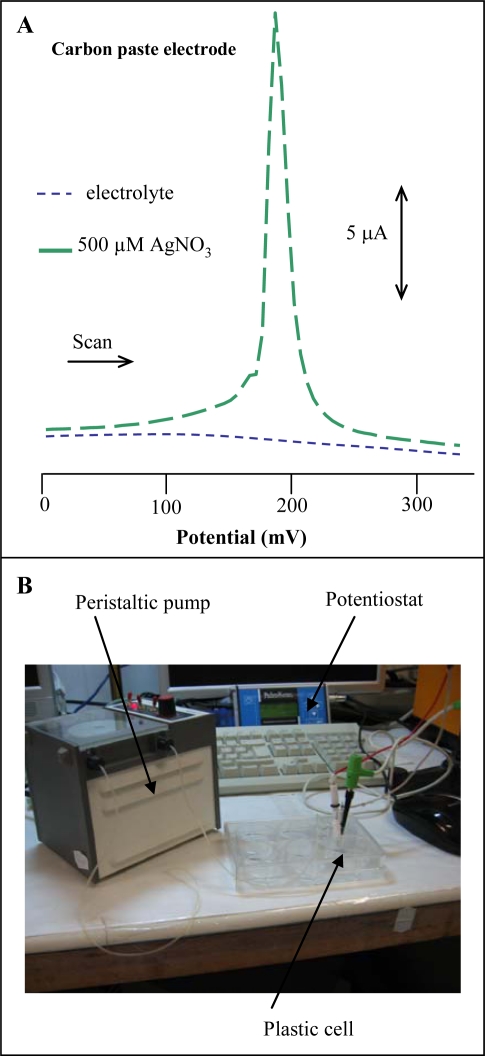
(A) DP voltammogram of 500 μM silver(I) ions concentration measured at carbon paste electrode. (B) Instrument consisted from potentiostat – PalmSens, plastic cell, peristaltic pump and three electrode system connected to the potentiostat (carbon tip or carbon pencil was used as working electrodes).

Several authors have reported on the use of carbon electrodes as the working one to determine silver(I) ions [24,26–29,35,36]. Thus, we utilized differential pulse voltammetry and CPE for silver ions determination. Electrochemical measurements were performed in the presence of 0.2 M acetate buffer (pH 5.0) at room temperature. At the surface of the carbon electrode silver(I) ions gave a signal of 200 mV with a relative standard deviation of 6.5%, n = 3 (Figure 3A). The detection limit (3 S/N) was estimated as 0.5 μM. In addition, repeatability of the results obtained with several carbon paste electrodes was good, depending on the paste used for filling the body of the electrode.

### 2.3. Microanalysis of Silver(I) Ions

**Figure 4.**
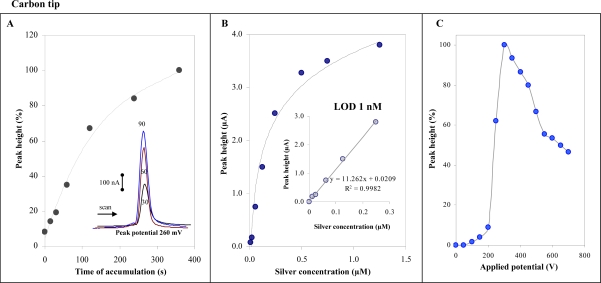
*Carbon tip working electrode.* (A) Dependence of peak height of silver(I) ions (1 μM) on the accumulation time measured at 0 V; in inset: DP voltammograms measured at accumulation times 30, 60 and 90 s. (B and inset) Dependences of peak height on silver(I) ions concentration (accumulation time 240 s, accumulation potential 0 V). (C) Hydrodynamic voltammogram of silver(I) ions. Flow rate of 0.2 M acetate buffer (pH 5.0) was 0.5 mL/min.

In the following experiments, we aimed at detection of silver(I) ions by using micro-instruments. For this purpose a miniaturized PalmSens potentiostat connected with and auxiliary platinum electrode and referent Ag/AgCl 3 M KCl electrode was utilized. As working electrode a carbon tip (1 mL) purchased from Eppendorf was used. Electrodes were placed in a simple plastic cell. Solutions exchange was carried out by peristaltic pump. The whole detection system was connected to a PC (Figure 3B). Measurements of silver(I) ions (1 μM) were performed in the presence of 900 μL 0.2 M acetate buffer (pH 5.0). We obtained well developed silver(I) ions signal at 260 mV at carbon tip (inset in Figure 4A). The height of signal was enhanced by increasing the time of accumulation up to 120 s, and then the signal enhanced more gradually (Figure 4A). The physico-chemical processes taking place at the carbon tip are not clear, it can be concluded that an interaction between the electrode surface and silver(I) ions takes place.

Further the calibration dependence was determined. Within a concentration range from 0 to 1.5 μM a characteristic non-linear calibration dependence was observed (Figure 4B). If we split the dependence we obtained a strictly linear curve within the range from 10 to 250 nM (inset in Figure 4B). Relative standard deviation was 5%. Limit of detection estimated by diluting of standard was 1 nM (time of accumulation 240 s). Due to the use of carbon tips in flow analysis a hydrodynamic voltammogram was determined. The potentials between 0 and 800 mV was applied to the working electrode and the response was determined (Figure 4C). The highest signal was measured at 300 mV.

### 2.4. Carbon Pencil

**Figure 5.**
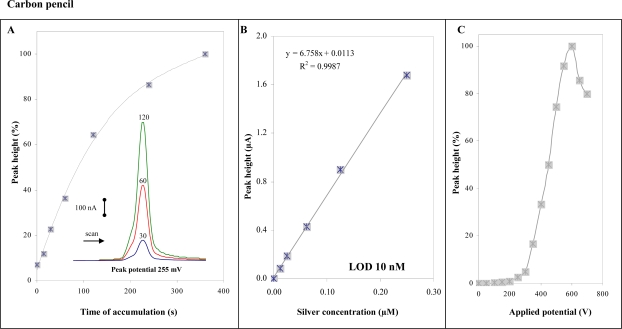
*Carbon pencil working electrode.* (A) Dependence of peak height of silver(I) ions (1 μM) on the accumulation time measured at 0 V; in inset: DP voltammograms measured at accumulation times 30, 60 and 120 s. (B and inset) Dependences of peak height on silver(I) ions concentration (accumulation time 240 s, accumulation potential 0 V). (C) Hydrodynamic voltammogram of silver(I) ions. Flow rate of 0.2 M acetate buffer (pH 5.0) was 0.5 mL/min.

In addition to detection of silver(I) ions at carbon tip, a carbon pencil (500 μm) was used for the same purpose. Using this electrode we obtained well developed silver(I) ions signal at 255 mV (inset in Figure 5A), which was enhanced with increasing time of accumulation up to 200 s, then the signals decreased (Figure 5A). Calibration dependence was measured within the concentration interval from 15 to 250 nM. The dependence was strictly linear with a regression equation y = 6.758x + 0.0113 and R^2^ = 0.9987. Relative standard deviation was 4.5%. Limit of detection estimated by diluting was 10 nM (time of accumulation 240 s). Course of hydrodynamic voltammogram for silver(I) ions measured at carbon pencil differed slightly compared to the carbon tip one, with a maximum at 600 mV (Figure 5C). The shift in potential can be related to different composition of the electrodes relating to interactions of silver (I) ions with a working electrode surface.

### 2.5. Silver(I) Ion Detection in Waters

We employed flow injection analysis coupled with a carbon tip as the most sensitive method to detect silver(I) ions released into various waters. During first, second and third week the amount of silver(I) ions released into water samples was under detection limit of the technique (Table 1). In the fourth week of the experiment the average concentration of silver(I) ions released to water samples was 7 ± 3 nM. The lowest concentration (3 nM) was determined in tap water, the highest in Regenia (12 nM). Silver(I) ions concentration increased during the twelve week long experiment, except for the concentration determined in tap water in the last week, where the marked decrease was observed. This phenomenon probably relates with the formation of water-insoluble silver compounds with salts (mostly Cl^−^) presented in tap water. At the end of the experiment the Ag(I) concentration was several times higher to the beginning of the release detected in the third week (Table 1). In addition, we observed marked changes in colour of plates at the end of the experiment.

### 2.6. Influence of Silver(I) Ions on Tobacco BY-2 Cell Suspension Culture

**Figure 6.**
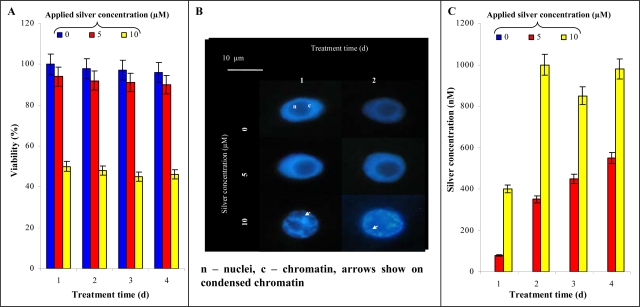
*Tobacco cells.* (A) Changes in viability of tobacco BY-2 cells treated with 0, 5 and 10 μM silver(I) ions for 4 days. (B) The influence of silver(I) ions on nuclei and chromatin condensation. (C) The content of silver(I) ions in the treated tobacco cells.

In the next experiments the influence of silver(I) ions on a plant model system (tobacco BY-2 cells) was investigated. Primarily we aimed our attention on the viability of the cells measured by double staining. Control BY-2 cell viability did not change during the four day long experiments and ranged between 97 and 100%. If BY-2 cells were exposed to silver(I) ions (5 μM), only a slight decline in viability was determined. Two times higher concentration caused a marked decrease in the viability already from the beginning of the experiment (Figure 6A). The structure and nuclear architecture of BY-2 cells treated with silver(I) ions was investigated, with emphasis on typical features of programmed cell death (PCD), such as cytoplasm shrinkage and chromatin condensation. The nuclei of untreated cells were predominantly regular, rounded within features of chromatin condensation. Application of 5 μM silver(I) ions didn’t lead to both viability and nuclear (chromatin) architecture changes. Contrary to this 10 μM silver(I) ions caused chromatin condensation (30–40% of all BY-2 cells), which it is one of the most typical and visible feature of PCD (Figure 6B).

Due to complexity of the extracts prepared from untreated and treated BY-2 cells, samples were analysed using HPLC coupled with a CoulochemIII detector to quantify silver(I) ions [29]. It follows from the results obtained that the BY-2 cells are able to accumulate silver(I) ions. During first and second day of the treatment the gradual enhancing of silver(I) ions content with increasing applied concentration of these ions was determined. Further increase of silver(I) ions content in BY-2 cells was more moderate (Figure 6C). This phenomenon probably relates with triggering of cells’ protective mechanisms against heavy metals [39,40].

### 2.7. Influence of Silver(I) Ions on Guppies

**Figure 7.**
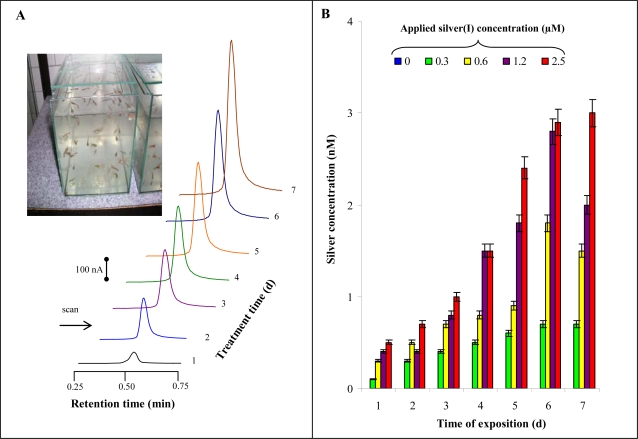
*Guppies.* (A) HPLC-ED chromatograms of silver(I) ions detected in tissues of fish treated with 2.5 μM silver(I) ions; in inset: guppies in tanks. (B) The content of silver(I) ions in treated guppies.

Silver(I) ions are very toxic to fish, hence their monitoring is very important indicator of environmental quality. In our experiments guppy fish were used. They were kept in 100 L aquaria according to OECD guidelines. In spite of the fact that all fish were fed, treated fish lost weight compared to untreated ones. At the very end of the experiment the weight of fish treated with 0.6, 1.2 and 2.5 μM of silver(I) ions was significantly lower compared to controls. HPLC-ED chromatograms of silver(I) ions detected in tissues of fishes treated with 2.5 μM are shown in Figure 7B. Content of silver(I) ions was enhanced with increasing applied concentration of these ions and time of treatment (Figure 7C).

## 3. Material and Methods

### 3.1. Chemicals, Materials and pH Measurements

Urease EC 3.5.1.5 (Jack Beans, type III; 45,000 IU/g) was purchased from Sigma Aldrich (St. Louis, MO, USA). Silver nitrate and all other reagents used were purchased from Sigma Aldrich (ACS purity unless noted otherwise). Stock standard solutions were prepared with ACS water (Sigma-Aldrich) and stored in the dark at a temperature of −20 °C. Working standard solutions were prepared daily by dilution of the stock solutions. All solutions were filtered through a 0.45 μm Nylon filter discs (MetaChem, Torrance, CA, USA) prior to HPLC analysis. The pH value was measured using WTW inoLab Level 3 with terminal Level 3 (Weilheim, Germany), controlled by the personal computer program (MultiLab Pilot; Weilheim, Germany). The pH-electrode (SenTix-H, pH 0–14/3M KCl) was calibrated by set of WTW buffers (Weilheim, Germany). Deionised water underwent demineralization by reverse osmosis using the instruments Aqua Osmotic 02 (Aqua Osmotic, Tisnov, Czech Republic) and then it was subsequently purified using Millipore RG (Millipore Corp., USA, 18 MΩ)–MiliQ water.

### 3.2. High Performance Liquid Chromatography with Electrochemical Detection (HPLC-ED)

The system consisted of a solvent delivery pump operating in range of 0.001–9.999 ml/min (Model 582 ESA Inc., Chelmsford, MA, USA), a guard cell (Model 5020 ESA, USA), a reaction coil (1 m) and/or a chromatographic column (Polaris C18-A, 4.6 mm, 5 μm particle size), and an electrochemical detector. The electrochemical detector (ED) includes one low volume flow-through analytical cell (Model 5040, ESA, USA), which is consisted of glassy carbon working electrode, palladium electrode as reference electrode and auxiliary carbon electrode, and Coulochem III as a control module. The sample (5 μL) was injected manually. The obtained data were processed by CSW 32 software. The experiments were carried out at room temperature. Guard cell potential was 0 V. A glassy carbon electrode was polished mechanically by 0.1 μm of alumina (ESA Inc., USA) and sonicated at room temperature for 5 min using a Sonorex Digital 10 P Sonicator (Bandelin, Berlin, Germany) at 40 W [29,41].

### 3.3. Electrochemical Measurement with Standard Potentiostat

Electrochemical measurements were performed using an AUTOLAB analyser (EcoChemie, The Netherlands). The three electrode system consisted of a carbon paste working electrode, an Ag/AgCl/3 M KCl reference electrode and a carbon counter electrode. The differential pulse voltammetry (DPV) parameters were as follows: initial potential −0.4 V, end potential 0.8 V, modulation amplitude 25 mV and step potential 0.5 mV. All experiments were carried out at laboratory temperature. Acetate buffer (0.2 M, pH 5.0) was used as the supporting electrolyte. The raw data were treated using the Savitzky and Golay filter (level 2) and the moving average baseline correction (peak width 0.03) of the GPES software. The carbon paste was made of 70% graphite powder (Aldrich) and 30% mineral oil (Sigma-Aldrich; free of DNase, RNase and protease). The carbon paste was housed in a Teflon body having a 2.5 mm diameter of active disk surface. The electrode surface was polished before each determination with a soft filter paper prior to measurement [42–45].

### 3.4. Microanalysis of Silver(I) Ions

Electrochemical measurements were performed using a PalmSens instrument (Palm Instruments BV, The Netherlands) using a plastic cell with three electrodes. The three electrode system consisted of carbon tip or carbon pencil working electrode, an Ag/AgCl/3 M KCl reference electrode and a platinum counter electrode. Carbon tips were purchased from Tosoh (Japan). A pipette tip is made from polymeric material and coated by graphite. The sampling tip is made of conductive resin and a part of the electrode of a capacitor which is a component of the oscillation circuit. The pencil leads (a lead diameter = 500 μm, total length = 60 mm) were purchased from Kohinor (České Budějovice, Czech Republic). Immersing 3 mm of the pencil lead into a solution resulted in an active electrode area of 4.91 mm^2^. The electrodes were used without any pre-treatment. They were polished mechanically by 0.1 μm of alumina (ESA Inc., USA) [46–48]. All other experimental parameters are same as in paragraph 3.3.

### 3.5. Silver Release Experiment–Water Samples

To study the releasing of silver ions into water, tap water from city of Brno, distilled water, Milli-Q water, common mineral waters (Mattoni, Aquila, Dobrá voda, Magnesia, Rajec), flavoured mineral waters (Aquila lemon, Regenia grapefruit) and beverages (Coca cola, Ice tea) available in the supermarket were used. Into one litre of certain water sample silver plate (app. 1 g) was added. During 13 weeks long experiment one millilitre was sampled per a week from certain sample of water.

### 3.6. Tobacco BY-2 Cell Suspension Culture

The suspension culture of *Nicotiana tabacum* BY 2 line was grown in liquid Murashige and Skoog medium supplemented with sucrose (30 g/L), KH_2_PO_4_ (0.2 g/L), thiamine (1 mg/L) and 2,4-dichlorophenoxyaetic acid (0.2 mg/L) according to Nagata [49]. The suspension cultures (20 mL) were grown in 50 mL Erlenmeyer flasks at 27 °C with shaking at 135 rpm (Kuhner Shaker, type: LT W, Adolf Kuhner AG, Switzerland). Subcultivation was performed after 3 or 4 days by transferring 2 or 1 mL, respectively, of suspension culture into a fresh medium (total volume 20 mL). After subcultivation cells were treated with three concentrations of silver(I) ions (0, 5 and 10 μM) for four days.

### 3.7. Double Staining

Modified double staining with fluoresceine diacetate (FDA) and propidium iodide (PI) for the determination of the viability of the cells was used [50,51]. In our experiments BY-2 cells (∼1 mg) were harvested and diluted with water, final volume of the mixture BY-2 cells+water was 50 μL. The stock solutions of PI and FDA were added to a final concentration of 20 μg/mL and 1 μg/mL respectively. After 5 min of incubation at room temperature, the percentage of dead (red-stained cells) and viable cells (green-stained cells) was evaluated using an Olympus AX 70 fluorescence microscope with an Olympus cube U MWU coupled with the digital camera. The percentage quantification of red (dead cells) and green areas (viable cells) was determined in acquired digital picture by method IA (Image–Pro Plus was used, ver. 1.3, Sony).

### 3.8. Chromatin Staining

Tobacco BY-2 cells were fixed by mixing of cell suspension with PEM buffer, ratio 1:1. PEM buffer consisted from 100 mM piperazine-1,4-bis(2-ethansulfonic acid) pH 6.9, 10 mM ethyleneglycol-bis(2-aminoethylether)-*N,N,N′,N′*-tetraacetic acid, 10 mM MgCl_2_ containing 4% formaldehyde (v/v). After 30 minutes long fixation the cells were three times washed by PEM buffer. The cells were resuspended by PEM buffer containing 0.1% of Triton X100 (v/v) and 1 μg/ml Hoechst 33285. Cells were observed by fluorescent microscope AX 70 (Olympus, Prague, Czech Republic).

### 3.9. Automated Spectrometric Measurements–Effects of Silver(I) Ions on Activity of Urease

Spectrometric measurements were carried using an automated chemical analyser BS-200 (Mindray, China). Reagents and samples were placed on cooled sample holder (4 °C) and automatically pipetted directly into plastic cuvettes. Incubation proceeded at 37 °C. Mixture was consequently stirred. The washing steps by distilled water (18 mΩ) were done in the midst of the pipetting. Apparatus was operated using software BS-200 (Mindray, China). The reagents for spectrometric measurements of urease activity were prepared as described in [10,52,53]. Solution of soya beans urease (10 μL) was mixed with 448 μL of hypochlorite solution (12% NaOCl, 0.4 M Na_2_HPO_4_ and 0.37 M NaOH, adjusted to pH 12) and with 42 μL of phenol solution (sodium nitroprusside, 7% phenol). This mixture was stirred and incubated for 15 min at 37°C. After this incubation the differences of absorption at 630 and 670 nm were measured. To investigate the effect of silver(I) ions on urease activity, solution of silver(I) ions (1 μM) was added to solution of soya beans urease.

### 3.10. Guppies

Guppies (*Poecilia reticulate*), 2 or 3 months old, were exposed to silver nitrate, always seven specimens per dose 0, 0.3, 0.6, 1.2, 2.5 or 5 μM. The experiment lasted 7 days (168 hours); a fish from each experimental variant was sampled per day. The experimental conditions such as pH value of the solution were kept constant; oxygen concentration and temperature were monitored during the experiment. Oxygen concentration varied within the range from 1.7 to 4.0 mg/L, the pH level from 6.34 to 7.00, and the temperature from 20.2 to 21.5 °C during the 7 days long experiment. The sampled fish was killed by CO_2_, washed one time with distilled water and one time with 0.5 M EDTA.

### 3.11. Descriptive Statistics and Estimation of Detection Limits

Data were processed using MICROSOFT EXCEL® (USA). Results are expressed as mean ±S.D. unless noted otherwise. Differences with p < 0.05 were considered significant (t-test was applied for means comparison). The detection limits (3 Signal/Noise ratio, 3 S/N) were calculated according to Long and Winefordner [54], whereas N was expressed as standard deviation of noise determined in the signal domain unless stated otherwise.

## 4. Conclusions

Suggesting of instruments for *in situ* analysis of target molecules is very topical. Heavy metals still are among the most dangerous environmental pollutants. In this work we show that a common carbon tip or carbon pencil coupled with a miniaturized potentiostat can be used for detection of silver(I) ions at nM levels. The instrument was tested on analysis of various water samples contaminated by silver(I) ions. Moreover, we used a commercial instrument liquid chromatography with electrochemical detection to analyse extracts from tobacco BY-2 cells and guppies treated with these toxic ions. If we consider selectivity of liquid chromatography and sensitivity of instrument proposed by us with working electrode as carbon tip, a small, portable, low cost and easy-to-use instrument could be fabricated.

## Figures and Tables

**Table 1.** Silver(I) ions concentration (nM) determined in waters during 13 weeks long experiment.

**Water sample**	Length of experiment (weeks)^**^										
	1–3^*^	4	5	6	7	8	9	10	11	12	13
Distilled water	nd	6	10	16	22	25	25	26	28	29	31
Tap water	nd	3	6	15	30	32	43	66	67	69	40
Coca cola	nd	11	15	29	32	33	34	37	38	39	40
Mattoni	nd	7	9	16	20	20	24	28	30	32	35
Rajec	nd	5	10	18	22	27	40	42	44	44	46
Regenia	nd	12	18	19	22	25	28	38	40	42	43
Ice tea	nd	9	15	18	21	21	28	36	38	38	40
Aquilla	nd	6	16	20	21	21	25	25	26	28	28
Milli-Q water	nd	8	12	18	18	21	22	22	24	24	25
Magnesia	nd	7	14	18	20	21	23	26	27	29	38

* Not Detected, no release of silver ions was observed.

** Relative standard deviation did not exceed 10%.
